# Targeting RNA with Next‐ and Third‐Generation Sequencing Improves Pathogen Identification in Clinical Samples

**DOI:** 10.1002/advs.202102593

**Published:** 2021-10-23

**Authors:** Na Zhao, Jiabao Cao, Jiayue Xu, Beibei Liu, Bin Liu, Dingqiang Chen, Binbin Xia, Liang Chen, Wenhui Zhang, Yuqing Zhang, Xuan Zhang, Zhimei Duan, Kaifei Wang, Fei Xie, Kun Xiao, Wei Yan, Lixin Xie, Hongwei Zhou, Jun Wang

**Affiliations:** ^1^ CAS Key Laboratory of Pathogenic Microbiology and Immunology Institute of Microbiology Chinese Academy of Sciences Beijing 100101 China; ^2^ University of Chinese Academy of Sciences Beijing 100049 China; ^3^ Peking University Third Hospital Beijing 100191 China; ^4^ College of Pulmonary and Critical Care Medicine Chinese PLA General Hospital Beijing 100853 China; ^5^ Microbiome Medicine Center Department of Laboratory Medicine Zhujiang Hospital Southern Medical University Guangzhou Guangdong 510282 China

**Keywords:** direct RNA sequencing, metagenome, metatranscriptome, Oxford Nanopore Technology

## Abstract

Fast and accurate identification of microbial pathogens is critical for the proper treatment of infections. Traditional culture‐based diagnosis in clinics is increasingly supplemented by metagenomic next‐generation‐sequencing (mNGS). Here, RNA/cDNA‐targeted sequencing (meta‐transcriptomics using NGS (mtNGS)) is established to reduce the host nucleotide percentage in clinic samples and by combining with Oxford Nanopore Technology (ONT) platforms (meta‐transcriptomics using third‐generation sequencing, mtTGS) to improve the sequencing time. It shows that mtNGS improves the ratio of microbial reads, facilitates bacterial identification using multiple‐strategies, and discovers fungi, viruses, and antibiotic resistance genes, and displaying agreement with clinical findings. Furthermore, longer reads in mtTGS lead to additional improvement in pathogen identification and also accelerate the clinical diagnosis. Additionally, primary tests utilizing direct‐RNA sequencing and targeted sequencing of ONT show that ONT displays important potential but must be further developed. This study presents the potential of RNA‐targeted pathogen identification in clinical samples, especially when combined with the newest developments in ONT.

## Graphical abstract

Targeting ribonucleic acid (RNA), the authors develop meta‐transcriptomic‐based pathogen detection using next‐generation sequencing (mtNGS) with Illumina and third generation sequencing (mtTGS) using Oxford Nanopore Technology (ONT), and show they outperform the current DNA‐based approaches in efficiency and accuracy, covering wider range of pathogens including bacteria, viruses and fungi. Further, direct RNA sequencing with ONT shows promising advantages and warrants further investigation.

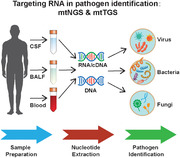

## 1 Introduction

Infection continues to be an immense threat to human health worldwide, casting a heavy burden on clinical diagnoses and treatments.^[^ ^1^, ^2^ ^]^ Pathogen identification in clinics is traditionally based on culture and biochemical assays but is increasingly complemented by metagenomic next‐generation sequencing (mNGS), which helps to identify difficult‐to‐culture organisms; in addition, identifying antibiotic resistance by sequencing also provides a basis for fine‐tuning treatment schemes.^[^ ^2^, ^3^, ^4^, ^5^ ^]^ However, mNGS still faces numerous obstacles in terms of its clinical application, especially when the materials used tend to be low in both volume and microbial quantity, such as throat swab samples, bronchoalveolar lavage fluid samples (BALF), blood samples, and cerebrospinal fluid samples (CSF). Simultaneously, host cells and thus nucleotides tend to constitute the majority in such samples (typically >90% host reads), dramatically reducing the efficiency of sequencing for microbial identification.^[^ ^6^, ^7^, ^8^ ^]^

Current platforms for next‐generation sequencing (NGS) are widely utilized for many applications, including pathogen identification, but they can only sequence DNA molecules and produce relatively short reads. While DNA composes the genetic material for pathogenic bacteria and fungi, RNA viruses compose a large fraction of infectious pathogens as well, including the influenza that causes seasonal flu as well as global pandemic and, at the time of this work, the severe acute respiratory syndrome coronavirus 2 (SARS‐CoV‐2) responsible for the coronavirus disease 2019 (COVID‐19) pandemic and global unrest.^[^ ^9^, ^10^, ^11^ ^]^ Thus, RNA viruses will be neglected from solely DNA‐based mNGS, sometimes hindering important diagnoses. Even for bacteria and fungi, despite relatively few studies on RNA‐based profiling or metatranscriptomics, the capability of revealing functionally active members/genes has provided novel insights into the human microbiome in host and health; in addition, while the bacterial genome has just a few copies of rDNA, rRNA molecules exist on a magnitude of thousands in active cells, and RNA targeting could, in theory, avoid dominant but dormant microbes making up a large percentage of sequencing reads.^[^ ^12^, ^13^, ^14^ ^]^

Interesting features from third‐generation sequencing (TGS), such as Oxford Nanopore Technology (ONT), might further improve sequencing‐based pathogen identification. NGS usually produces fragmented, short reads that need to be assembled before taxonomic assignments and/or antibiotic resistance determination, and the time required for sequencing (usually days) on current NGS platforms also takes a few days. However, shortening the diagnosis time can greatly benefit patients; for instance, intensive‐care unit (ICU) patients face life‐threatening infections, such as sepsis, encephalitis, meningitis, toxin‐induced cardiocirculatory shock, failures of central nervous system function, respiratory insufficiency or multiorganic failure, which can become lethal within hours; and consequently, their survival rate is estimated to increase 7% by each shortened hour for pathogen diagnosis.^[^ ^15^, ^16^, ^17^, ^18^ ^]^ ONT, in contrast, could produce longer reads (hundreds to thousands, or even longer base pairs) and generate sequences in real time, and it has already been recently applied in clinical applications for the rapid identification of pathogens targeting microbial DNA.^[^ ^19^, ^20^, ^21^ ^]^ In addition, ONT offers novel prospects for clinical application due to its capacity for direct RNA sequencing.^[^ ^22^, ^23^ ^]^ In view of these rationales, we carried out a prospective study of targeting RNA (thus metatranscriptome) for microbial pathogen profiling in representative clinical samples, demonstrating its improvement from DNA‐targeted sequencing approaches, and we further explored the potential of using the ONT platform for pathogen identification with either cDNA sequencing or direct RNA sequencing.

## 2 Results

### 2.1 Description of Samples Used in This Study

In this study, we collected 39 bronchoalveolar lavage fluid (BALF) samples, 14 blood samples, and nine cerebrospinal fluid (CSF) samples, each from different patients diagnosed with infections. Samples were obtained from two medical centers in Beijing and one in Guangzhou (**Table** **1** and Table S1 and Figure S1, Supporting Information). Patients were primarily elderly, and BALF samples were collected within the first 12 h of patients entering respiratory ICUs, while blood and CSF were obtained from patients suspected of infection in respective systems during clinical visits. First, a baseline metagenome and metatranscriptome of clinical samples was established without any enrichment of microbial materials or removal of host cells/nucleotides. Then mNGS using total DNA, and total RNA reverse‐transcribed into cDNA and sequenced (mtNGS) with the Illumina platform as well as ONT (meta‐transcriptomics using third‐generation sequencing, mtTGS) were carried out, while some RNA samples were used for direct RNA sequencing using ONT (Figure S2, Supporting Information).

**Table 1:** Summary of the clinical characteristics of the samples used in this study

Characteristic	Overall	BALF	Blood	CSF
**Age**				
Mean yr	59.8	66.2	34.5	25.4
**Distribution no. (%)**				
0–1 yr	7 (11.3)	0	2 (14.3)	5 (55.6)
1–20 yr	8 (12.9)	3 (7.7)	5 (35.7)	0
21–60 yr	17 (27.4)	10 (5.6)	4 (28.6)	3 (33.3)
≥60 yr	30 (48.4)	26 (66.7)	3 (21.4)	1 (11.1)
**Sex no. (%)**				
Male	50 (80.6)	33 (84.6)	11 (78.6)	6 (66.7)
Female	12 (19.4)	6 (15.4)	3 (21.4)	3 (33.3)

### 2.2 MtNGS Surpasses mNGS in Microbial Profiling Efficiency

**Figure 1:**
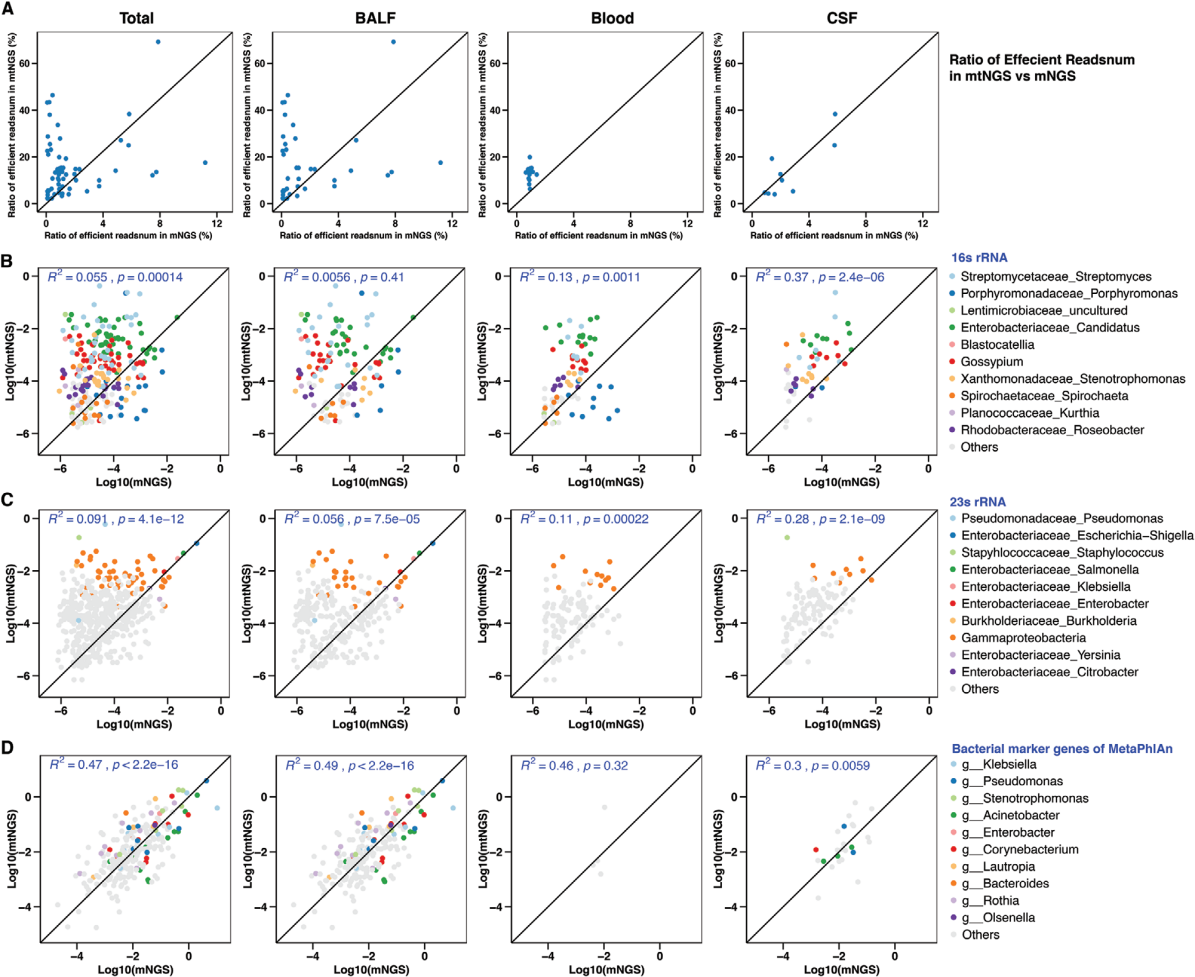
Sequencing efficiency regarding the proportion of microbial reads and correlation in bacterial abundances between mtNGS and mNGS. A total of 62 samples, including 39 BALF samples, 14 blood samples, and nine CSF samples, were available for comparison in mtNGS and mNGS sequencing. A) The proportion of microbial mapping reads in combined samples and each sample type separately in mtNGS and mNGS. Percentages were calculated by dividing by total reads. For BALF and blood samples, an overall increase in microbial read ratios was observed for mtNGS (above the 1:1 line, relative to mNGS samples). B–D) Correlations of shared bacterial species calculated using multiple gene markers across all samples, as well as in each sample type in mtNGS and mNGS. The relative abundance of bacterial was defined as the proportion of marker reads for the respective bacteria relative to all reads and correlations were calculated using log(10) transformed nonzero proportions. *R* ^2^ denotes the square of the pearson correlation coefficient calculated using the stat_cor function. Markers were B) the 16S rRNA gene, C) 23S rRNA genes, D) and MetaPhlAn marker genes. The top ten species in terms of relative abundance across samples are indicated by colored dots, while others are indicated in gray. Total: All sample types involved in our study; BALF: bronchoalveolar lavage fluid samples; Blood: blood samples; CSF: cerebrospinal fluid samples.

Based on our analyses (see the Experimental Section), it is first demonstrated the higher efficiency of mtNGS for microbial profiling in general, including a higher proportion of microbial reads than with the mNGS approach and, in particular, elevated copy numbers of taxonomy‐resolving genes (16S and 23S rDNA/rRNA genes for bacteria). Although host DNA was predominant in DNA‐based mNGS (>80% overall), microbial cDNA was significantly increased and enriched in the mtNGS results (2–69%, >sevenfold change on average) (**Figure** **1**A and Tables S2 and S3, Supporting Information). Second, within the microbial reads, it is also observed an elevated proportion of ribosome‐containing 16S rRNA and 23S rRNA, which are also commonly used for resolving bacterial taxonomy. While reads mapped to 16S rRNA reached 0.0014% (0–0.024547%) on average in mNGS (BALF: 0–0.02454%, blood: 0.000003–0.002246%, CSF: 0.00002–0.002695%), these ratios significantly increased to 0.349158% (≈250‐fold increase, 0.000324–9.318718%) on average across all samples (BALF: 0.000324–9.318718%, blood: 0.001861–0.119694%, CSF: 0.002231–2.305679%) in the mtNGS results. Similarly, for 23S rRNA, the relative abundance of reads on average in all samples reached 0.005672% (0–0.231169%) in mNGS (BALF: 0–0.231169%, blood: 0.000016–0.002798%, CSF: 0.000075–0.012011%) and increased to 0.874654% (≈154‐fold change, 0.000739–6.193371%) in mtNGS (BALF: 0.000739–4.826381%, blood: 0.003938–6.193371%, CSF: 0.006771–4.986741%) (Tables S4 and S5, Supporting Information). The data suggested that even without physical or chemical removal of host cells, using RNA/cDNA could dramatically decrease the interference and a high proportion of host nucleotides, lowering the efficacy of mNGS, and for bacterial profiling, higher copy numbers of rRNA per cell could also improve the detection of active members using a limited amount of materials/reads.

MtNGS‐resolved microbial compositions had considerable congruences with mNGS in the shared bacterial species. In terms of total bacteria, mNGS discovered only 0.55% of all bacterial species discovered by mtNGS using 16S rDNA/rRNA as a phylogenetic marker and 5.8% considering 23S rDNA/rRNA (Figure S3, Supporting Information). Using the relative abundances of 16S genes as surrogates for bacterial abundances, there were significant correlations for shared bacterial species across all samples (*r* ^2^ = 0.055, *p* < 0.05) and individually in blood (*r* ^2^ = 0.13, *p* < 0.05) and CSF samples (*r* ^2^ = 0.37, *p* < 0.05), while relatively more complex communities, such as BALF, showed low, nonsignificant correlations (*r* ^2^ = 0.0056, *p* > 0.4) (Figure 1B). Using 23S rDNA, significant correlations were found between shared bacterial abundances using all samples (*p* < 0.05) as well as in each individual type of sample (Figure 1C). Using MetaPhlAn, all microbial reads were further incorporated. Considering multiple phylogenetic markers for individual bacterial groups, MetaPhlAn was used to calculate the abundances of different bacterial species and showed that mNGS and mtNGS reached considerable correlations in terms of microbial profiling (*r* ^2^ > 0.3 in all samples and individual sample types, all *p* < 0.05 except in blood samples) (Figure 1D). Our results indicated that while mtNGS had improved efficiency, it did not lead to significant deviations in microbial profiling in comparison to mNGS.

### 2.3 MtNGS Improves Fungi, Virus, and Antibiotic Resistance Gene (ARG) Detection

**Figure 2:**
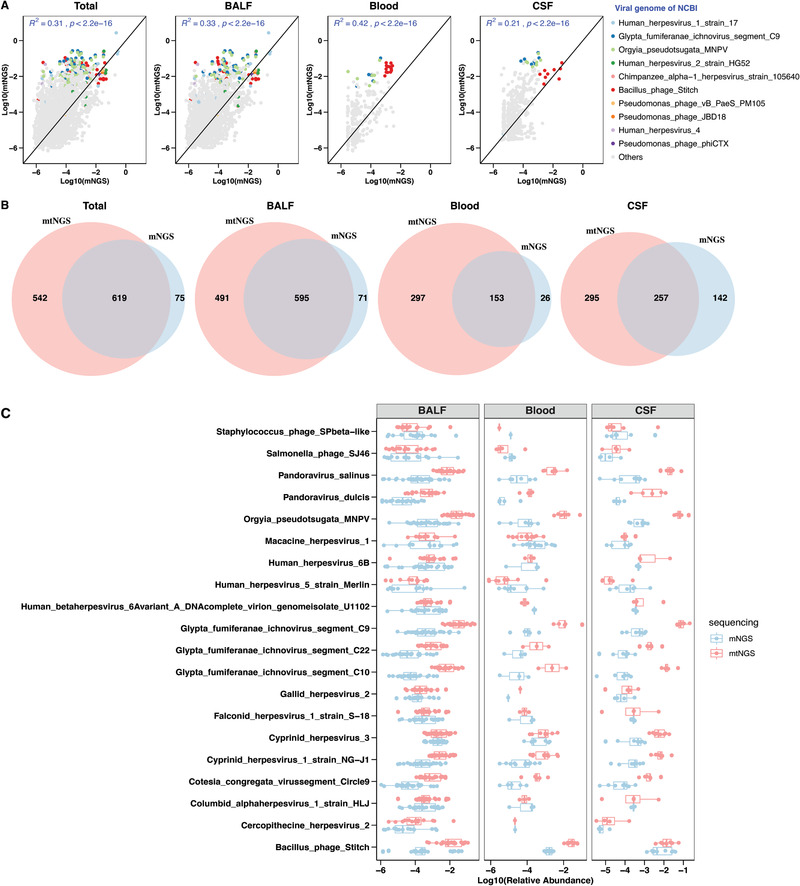
Improved viruses detection in mtNGS in comparison to mNGS. A) The correlation between the relative abundance of shared viruses using all samples and each individual type of sample in mtNGS and mNGS (*n* = 62 in each sequencing assay). The abundances of viruses were calculated by mapping against the viral genome from the NCBI viral genome database and the mapped number of reads was then divided into total reads and nonzero abundances were retained. *R* ^2^ and statistical significant were assessed using stat_cor function. The top ten viruses in terms of relative abundance across samples are indicated by colored dots, while others are indicated in gray. B) Venn diagram illustrating the overlap of the viral composition between mNGS and mtNGS. C) Distribution of relative abundances (log10‐transformed) of major virus groups in mtNGS and mNGS, showing approximately two‐magnitude higher viral reads in the results of mtNGS. Total: All sample types involved in our study; BALF: bronchoalveolar lavage fluid samples; Blood: blood samples; CSF: cerebrospinal fluid samples.

We then conducted virome profiling to identify viral pathogens critical for clinical diagnosis and found that mtNGS was effective in dissecting viral composition and identifying potential viral pathogens. By mapping to collective viral genomes, the mtNGS approach discovered 1161 viruses in all samples (1086 in BALF, 450 in blood samples, and 552 in CSFs), in contrast to 694 viruses using mNGS (666 in BALF, 179 in blood, and 399 in CSF). Importantly, 271 RNA viruses, including known infectious viruses such as hepatitis C virus (genotypes 1, 2, and 6), human coronavirus 229E, and human parainfluenza virus 3, were identified using mtNGS assay. In mtNGS and mNGS approach, 619 shared viruses were identified in all samples and their abundances showed significant correlations among different categories of samples (*r* ^2^ = 0.31 in all samples, 0.33 in BALF, 0.42 in blood and 0.21 in CSF samples, all *p* < 0.05). More importantly, the relative abundances of major virus groups were between one to two magnitudes higher in mtNGS than in mNGS, further demonstrating the higher efficiency in viral detection and profiling using mtNGS (**Figure** **2** and Tables S4 and S5, Supporting Information).

**Figure 3:**
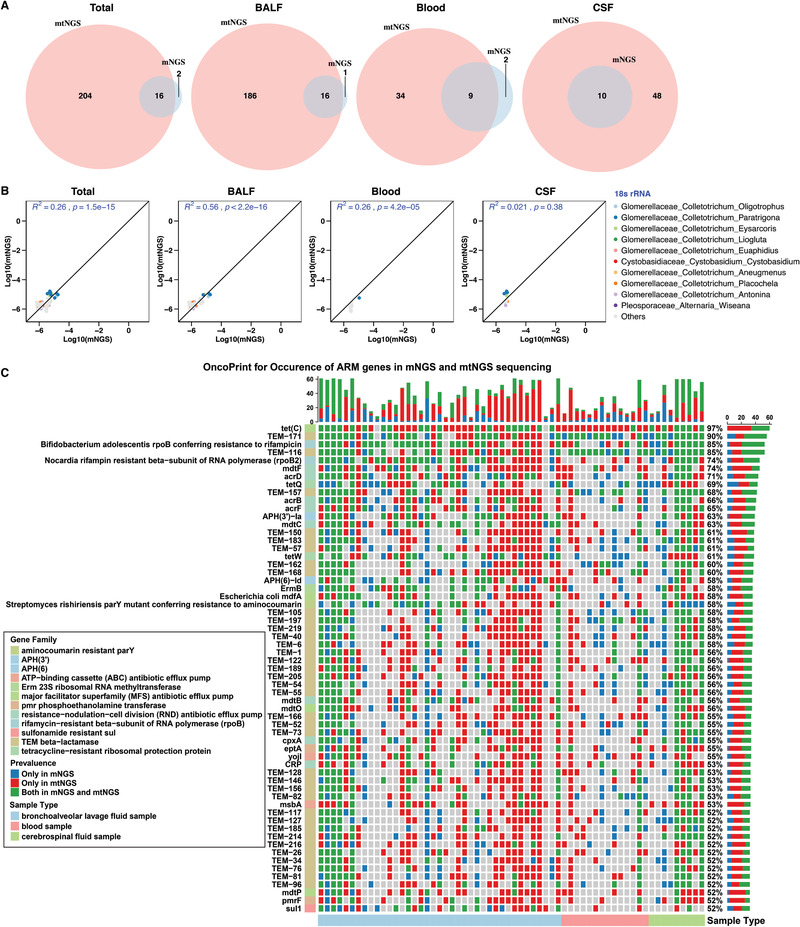
Improved fungi and ARGs detection in mtNGS compared with mNGS. A) Venn diagram illustrating the overlap of fungi composition between mNGS and mtNGS, showing that mtNGS discovers many more fungi and covers the majority of mNGS findings. B) The correlation between the nonzero relative abundance of shared fungi in all samples and each individual type of sample (*n* = 62 in each sequencing assay), calculated by the percentage of reads mapped to 18S rRNA genes divided by the total reads, followed by a log10 transformation. *R* ^2^ and statistical significant were assessed using stat_cor function. The top ten fungi in terms of relative abundance across samples are indicated by colored dots, while others are indicated in gray. Total: All sample types involved in our study; BALF: bronchoalveolar lavage fluid samples; Blood: blood samples; CSF: cerebrospinal fluid samples. C) Distribution of high‐occurrence ARGs (61 ARGs appearing in 52–97% of the samples) across all samples and three different sample types, detected with the mtNGS and mNGS methods. ARGs in blue were detected using mNGS only, red using mtNGS only, and green using shared detection in both methods. The top and right‐hand stacked bars represent the number of ARGs and the frequency in each individual type of sample, respectively.

Similarly, pathogenic fungi remained largely excluded from many mNGS applications despite being important causes of clinical infections as well as death, and again, we demonstrated the improved efficiency of mtNGS to detect fungi. In addition to bacteria, elevated copy numbers of the taxonomy‐resolving gene 18S rRNA in mtNGS were identified compared to mNGS. While in mNGS 18S commonly reached 0.000013% (0–0.000109%) on average (BALF: 0–0.000109%, blood samples 0–0.00003%, CSF: 0.000005–0.000051%), the relative ratio of 18S reads increased more than fivefold to 0.000071% on average (0.00001–0.000672% in all samples, BALF: 0.00001–0.000672%, blood: 0.00001–0.000071%, CSF: 0.000026–0.000551%) in mtNGS (Tables S4 and S5, Supporting Information). Simultaneously, mtNGS and mNGS reached significant correlations in terms of shared fungal species (r^2^ = 0.26 in all samples, 0.56 in BALF, 0.26 in blood, and 0.021 in CSF, all *p* < 0.05 except in CSF samples, **Figure** **3**A,B), showing a high congruence between mtNGS and mNGS for fungal profiling.

Widespread antibiotic resistance underlies persistent infections and constitutes a global health threat and identifying the spectrum of antibiotic resistance in clinical diagnosis is critical for determining treatment schemes.^[^ ^24^ ^]^ Thus, analysis was expanded to determine the efficiency of mtNGS in identifying ARGs. MtNGS and mNGS reads were mapped against the Comprehensive Antibiotic Resistance Database (CARD), and in mNGS, 1004 ARGs were identified in all samples (986 in BALF, 287 in blood, and 385 in CSF), while the mtNGS data also revealed 914 in all samples (885 ARGs in BALF, 190 in blood, and 347 in CSF), with high correlations between DNA and cDNA (*r* ^2^ = 0.33 in all samples, 0.41 in BALF, 0.023 in blood, and 0.084 in CSF) for shared ARGs. Although mtNGS did not differ greatly from mNGS in the total number of ARGs discovered, further inspection revealed the ARGs with higher occurrences (appearing in 52–97% of the samples, 61 ARGs in total), with mtNGS alone discovering 45.9% (1034/2250 occurrences) and mtNGS and mNGS providing a shared identification of 37.4% of such ARGs (838/2250 occurrences), while only 16.8% (378/2250 occurrences) were found by mNGS (Figure 3C). In short, mtNGS was more effective than mNGS in discovering high‐occurrence ARGs but not the whole ARG spectrum, likely due to its tendency to focus on highly active genes in response to antibiotic stress.

### 2.4 MtNGS Agrees Better with the Clinical Diagnosis Using Conventional Methods

To compare the ability of mNGS and mtNGS to support the clinical diagnosis using conventional methods (mainly culturing for bacterial pathogens), culture‐based results were crosschecked for the clinical samples used in this study with our mNGS and mtNGS data. Of all the samples subjected to mNGS and mtNGS (*n* = 62 in each assay), 42 had positive bacterial culture results, and a total of 61 strains of bacteria were identified. The majority were from BALF samples with 29 samples and 47 isolates, and the rest were from four blood samples with four isolates and nine CSF samples with ten isolates. The most common species were *Acinetobacter baumannii* (eleven strains), *Staphylococcus aureus* (eight strains), and *Klebsiella pneumoniae* (nine strains), and the rest of the bacterial species had fewer than five total isolates in our samples. Surprisingly, mNGS was only capable of reporting one strain of *Klebsiella pneumoniae* using 16S rDNA mapping and four strains of bacteria using 23S rDNA. Even by expanding the number of genetic markers using MetaPhlAn, the number of positive discoveries (i.e., identifying clinically isolated bacteria using mNGS) achieved only 26 (42.6% of all clinical isolates). In contrast, mtNGS significantly improved the positive discovery using single phylogenetic markers, identifying 25 strains using 16S rRNA (25‐fold increase compared with mNGS) and 40 using 23S rRNA (tenfold increase). In terms of the MetaPhlAn‐based approach using multiple markers, mtNGS achieved nearly the same amount of positive discovery (25), missing *Staphylococcus aureus, Streptococcus pneumoniae*, and *Escherichia coli* independently in three samples but simultaneously discovering *Escherichia coli* and *Enterococcus* independently in another two samples that were not detected by mNGS (Table S6, Supporting Information).

We then expanded the comparison of mtNGS versus mNGS to examine their power to discover pathogenic viruses and fungi, and it is found that despite the overall low number of viral/fungal pathogens diagnosed in clinics, the performance of mtNGS still surpassed that of mNGS. Viral pathogens were only identified in blood samples using a corresponding polymerase chain reaction (PCR) detection kit for clinically relevant viruses, with nine samples positive for either influenza A virus (six samples), hepatitis C virus (two samples), or human rhinovirus (one sample). Among those, mtNGS identified hepatitis C virus in two samples and one influenza A virus sample. Similarly, in terms of fungal pathogens, eight BALF samples were identified to have nine isolates of fungi, mainly *Candida albicans* (four samples) and *Saccharomyces* (two samples), using culture‐based methods. Here, only positive identification was achieved with mtNGS for *Saccharomyces* using 18S rRNA as a genetic marker, and mNGS could not positively identify any fungal pathogens.

As for the abundance of sequencing in each sample, MetaPhlAn (multimarker) approach showed that the bacterial pathogens detected by conventional methods were among the most abundant taxa discovered in the mNGS (median rank 2nd) and mtNGS (median rank 3rd), while depending on single markers such as 23S still identified the corresponding bacteria among the top ten species (median rank 8th for mNGS and 7th for mtNGS, respectively), though using 16S marker only revealed one positive identification in mNGS, and low ranking for pathogens in mtNGS. (Table S6, Supporting Information)

### 2.5 ONT‐Based mtTGS of cDNA for Pathogen Profiling

After establishing that mtNGS could improve pathogen identification compared with mNGS on several fronts, we then applied cDNA sequencing using ONT MinION platforms (mtTGS). ONT allows relatively quick library preparations of DNA libraries within hours, and combined with real‐time sequencing and base‐calling on an individual‐read basis, these features have already been used to accelerate the clinical diagnosis of pathogens using DNA.^[^ ^19^, ^20^ ^]^ In 43 samples, mtTGS generated 73–550 Mb total nucleotides per sample, representing nearly 0.7–5.9% of the corresponding mtNGS output (Table S7, Supporting Information).

**Figure 4:**
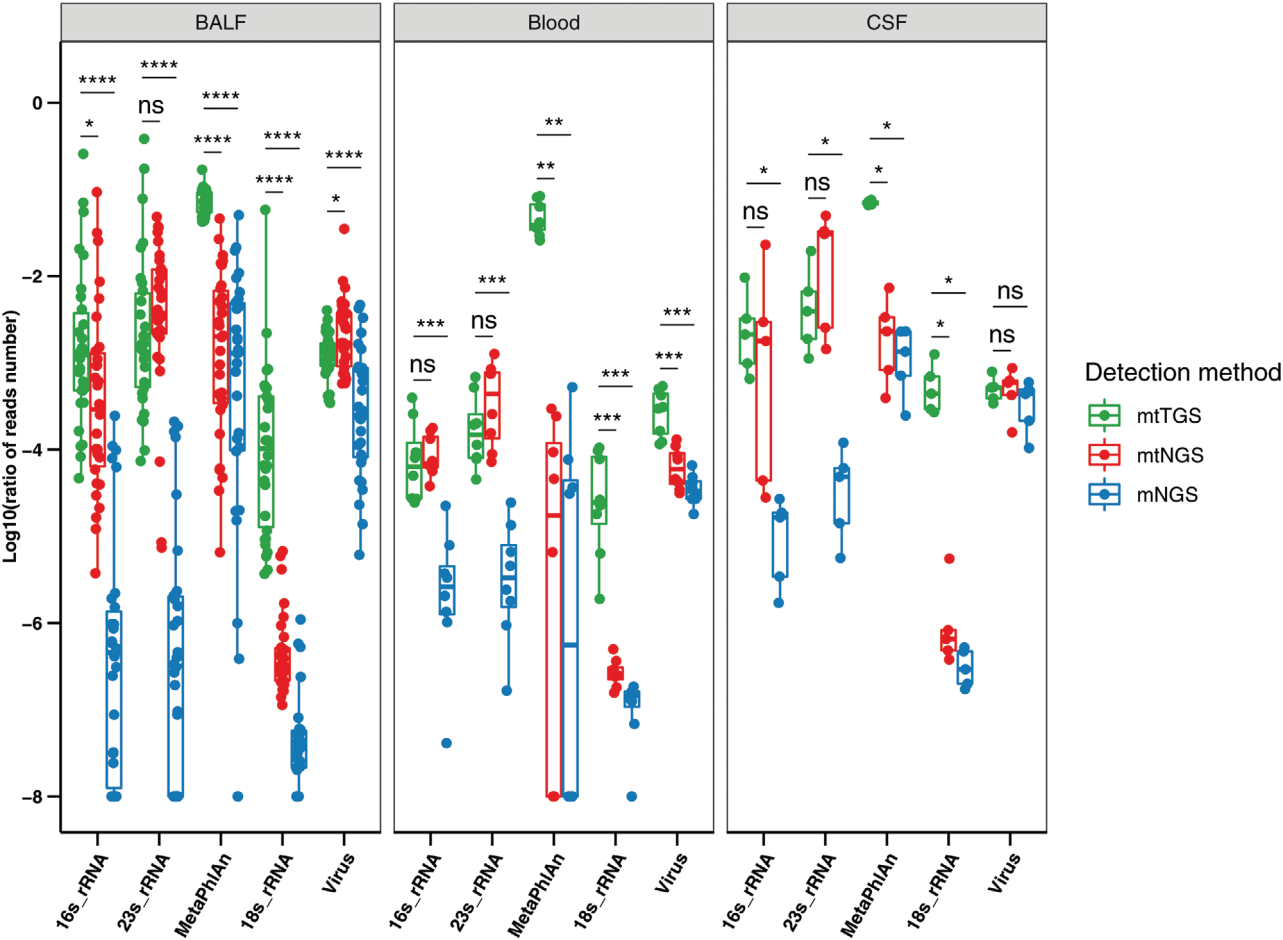
mtTGS increased the mapping rate for major markers used for bacteria, fungi, and virus identifications. The percentage of microbial mapping reads (bacteria, fungi, and viruses) was calculated in each individual type of sample (*n* = 43) for mtTGS (green), mtNGS (red), and mNGS (blue). MtTGS had an overall approximately two‐magnitude higher percentage of microbial reads than mNGS, as well as a higher percentage of 18S/MetaPhlAn‐markers than mtNGS. Significant differences in pairwise comparisons between mtTGS versus mtNGS and between mtTGS versus mtNGS are shown. Statistical significant was assessed with Wilcoxon rank‐sum test. *P  <  0.05, **P  <  0.01, ***P  <  0.001, ****P  <  0.0001, ns, not significant; BALF: bronchoalveolar lavage fluid samples; Blood: blood samples; CSF: cerebrospinal fluid samples.

Microbial compositional analyses revealed further improvements in mtTGS in terms of sequencing efficiency. Compared with mtNGS, mtTGS provided similar percentage of microbial reads, and both were higher than mNGS. However, the percentage of reads that could be mapped to fungi (18S) increased significantly in all three sample types, as well as the total reads that could be mapped to the database used in MetaPhlAn for bacteria identification, all with a high fold change (for 18S, median fold change of 313.33; for MetaPhlAn, median fold change of 80.79) (**Figure** **4**). Potentially, this improvement was due to the longer length of ONT reads (average reads length of 334.9–539.1 bp, in contrast to mNGS/mtNGS having 150 bp, paired‐end reads) to improve overall mapping rates to reference databases.

When comparing the identity of the identified bacteria and fungi, it is found that the highest number of shared microbes discovered by more than two methods (out of three: mtTGS, mtNGS and mNGS) were between mtTGS and mtNGS using 16S rRNA, 23S rRNA, or 18S rRNA markers, demonstrating a high agreement between metatranscriptomic‐based methods on different platforms. Additionally, for bacteria identified by MetaPhlAn and viruses, the highest number of shared taxa were discovered by all three methods, revealing an overall high accordance between sequencing methods when using multiple markers or genome sequences. However, this agreement could not be established for ARG genes, as mtTGS identified only 0.0056–13% of ARG genes identified in mtNGS. We speculate that this was due to the lower number of mtTGS reads (only 0.2–15% of those in mtNGS) as a result of the relatively low output of the ONT platform, preventing the discovery of low‐abundance genes.

Finally, crosschecking the previously mentioned clinical diagnosis and identification by mtTGS confirmed that the higher mapping rate against the MetaPhlAn database further increased the positive discovery rate compared with that of mtNGS. In a total of 28 samples with bacterial culture and mtTGS results (20 BALF, three blood, and five CSF), 43 isolates of bacteria belonging to 19 species were identified, in which mtTGS managed to positively discover 34 using 16S rRNA, 36 using 23S rRNA, and 37 using MetaPhlAn, showing a considerable increase from 22, 34, and 19 in mtNGS, respectively. It is attributed that this improvement to the higher read length and consequently higher mapping rates to reference databases, facilitating the positive identification of reads belonging to those pathogens. As to the sequences abundance in each sample, mtTGS results showed increased detection even using single markers such as 16S and 23S, and improvement in ranking of the main pathogens (ranked 5.5th and 8th in median, respectively), as we attributed to increased sequence lengths; but seemingly using multimarker (MetaPhlAn) is slightly problematic as the ranks of key pathogens are low (median 20th), which could result from the high error rate of ONT reads and consequently misidentifications on individual marker genes. In discovering of pathogenic fungi, mtTGS showed increased detection by using 18S markers (ranked 6.5th in mtTGS results and 13th in mtNGS, in median). While in pathogenic virus detection, mtNGS was also more sensitive than those two approaches, mNGS and mtTGS (Table S8, Supporting Information).

### 2.6 Direct RNA Sequencing Using ONT Platforms of Clinical Samples

In contrast to mtNGS based on the Illumina platform, ONT offers the capacity to directly sequence native RNAs without the need for prior cDNA synthesis and PCR amplification. Direct RNA sequencing then was applied using ONT MinION platforms, which in theory could reduce the biases caused by cDNA synthesis and greatly improve the sequencing speed of RNA from microbes. However, direct RNA sequencing currently faces the challenge of a relatively low amount of total RNA in clinical samples, and only obtained a reasonable amount of direct RNA sequencing library (i.e., detectable concentration of RNA after library preparation) and sequencing results from 11 samples, including eight BALF, two blood samples, and one CSF sample, resulting in 12–11 500 kb total nucleotides in each sample (Table S9, Supporting Information). The low output in direct RNA sequencing was largely due to the low amount of starting RNA from clinical samples and partially also due to the efficiency in library preparation as well as sequencing on the ONT platform at this stage.

**Figure 5:**
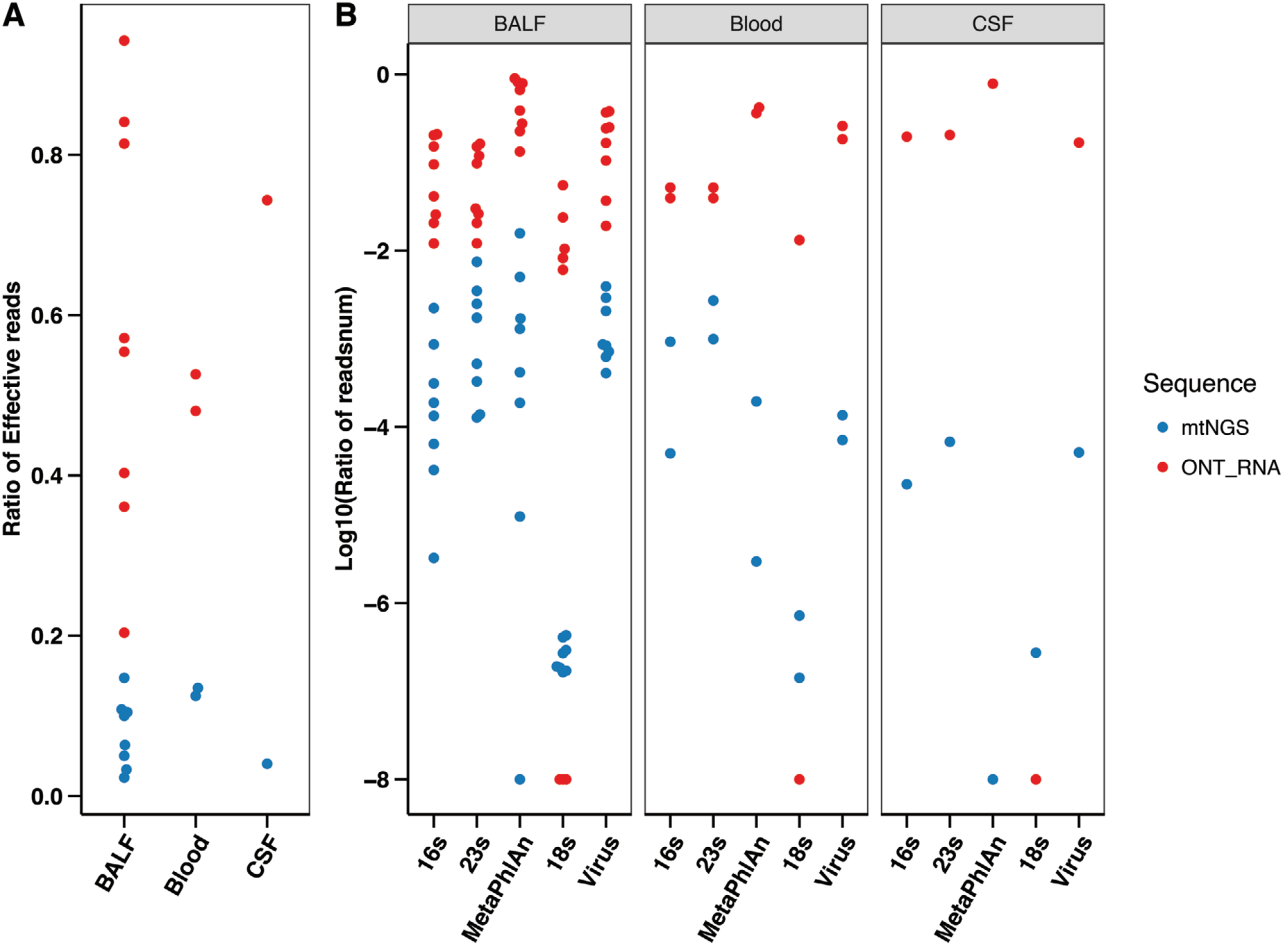
Direct RNA sequencing further increased the mapping rate percentage. A) The proportion of microbial mapping reads in each individual type of sample (*n* = 11) using the mtNGS and ONT direct RNA platforms. B) The percentage of microbial mapping reads (bacteria using 16S, 23S, and MetaPhlAn markers, respectively; fungi using 18S; and viruses using reference genomes) was calculated in each individual type of sample (*n* = 11) by mtNGS and ONT direct RNA sequencing, followed by log10 transformed. BALF: bronchoalveolar lavage fluid samples; Blood: blood samples; CSF: cerebrospinal fluid samples.

Despite the low output in direct RNA sequencing, several characteristics of RNA sequencing results still present promise for future application and improvement in microbial profiling and clinical diagnosis. Overall, direct RNA results had a higher proportion of microbial reads than those resulting from mtNGS; in BALF, the ratio reached as high as 94.3% and, on average, 57.4%, with a 3.76–15.69‐fold increase from mtNGS. Additionally, in blood and CSF, we observed 3.57–4.22‐ and 18.49‐fold increases, respectively. It is not yet clear whether the differences were from biases introduced into cDNA during reverse transcription and amplification or the bias of direct RNA sequencing toward microbial reads. The higher proportion of microbial reads also led to an overall higher ratio of reads mapped to 16S, 23S, and MetaPhlAn markers and 18S and viral genomes in each sample type. A significant increase was observed in the percentage of reads that could be mapped to bacteria (fold change for 16S: 29.7–29374.3; fold change for 23S: 19.1–3036.4; fold change for MetaPhlAn: 50.5–142549.7), fungi (fold change: 0–305984.484), and viruses (fold change: 17.8–3663) (**Figure** **5** and Table S9, Supporting Information) in the direct RNA sequencing results. However, in terms of the total number of bacteria, fungi, and viruses identified, direct RNA sequencing results had a much lower number due to the low number of reads (Table S9, Supporting Information).

Eventually, direct RNA sequencing was extended for diagnosis and genomic analysis of SARS‐CoV‐2 virus in COVID‐19 patients and demonstrated that direct RNA could be used for the diagnosis of this important RNA virus. In a total set of two swabs and seven fecal samples that were confirmed to be SARS‐CoV‐2 positive with real‐time reverse‐transcription PCR (RT‐PCR), direct RNA sequencing with ONT generated 14–1474 Mb sequences (Table S10, Supporting Information). Reads mapping to the SARS‐CoV‐2 reference genome were all present but with relatively low numbers (one read to 117 reads, corresponding to 1.6 × 10^–6^–1.8 × 10^–5^%), and we managed to establish a considerable positive correlation of the read number (*R* = 0.83, *p* = 0.008) to that of the viral load as determined by RT‐PCR (Figure S4, Supporting Information). Limited by the total number of reads from RNA combined with the extremely low abundance of SARS‐CoV‐2, performing in‐depth genomic analysis of this virus could not be performed. However, this limitation has been reported even for more established methods using Illumina sequencing, in which targeted amplification of the SARS‐CoV‐2 virus genome is necessary to achieve appropriate coverage of the genome for further analysis.

### 2.7 Further Removal of Host cDNA by Targeted Sequencing Using ONT

In addition, to compensate for the relatively low output from cDNA (and potentially direct RNA sequencing) using the ONT platform, targeted sequencing was applied by further increasing the percentage of microbial reads. ONT is able to utilize a “read‐until” strategy in which when reads with partial sequences match a defined database, the electric current in the corresponding sequencing nanopore can be reversed and sequencing terminates for particular reads. Using the host genome and transcriptome as a reference to remove part of the host cDNA reads from ONT cDNA sequencing might further improve the percentage of microbial cDNA in the final sequencing results. Thus, experimenting with two recent developments in targeted sequencing based on ONT, Readfish,^[^ ^25^ ^]^ and UNCALLED,^[^ ^26^ ^]^ and four samples were included to examine the performance of targeted sequencing for mtTGS.

Compared with the ONT mtTGS results (with an average of 93.97% host reads), it is observed that detectable but small decreases in host reads during targeted cDNA reads by Readfish (average of 93.15% host reads). UNCALLED, on the other hand, resulted in an average of 96.76% host reads in the sequencing results (Figure S5, Supporting Information). Thus it is concluded that at this stage, such a “read‐until” strategy could not yet significantly improve cDNA sequencing, potentially because the two methods were originally developed for long read sequencing, and during mtTGS sequencing, there is insufficient time between identifying targeted reads and consequent removal for relatively short reads of cDNA (only hundreds of bp). As a result, such a strategy might need further improvements in computational methods as well as hardware before its effective application in clinical samples and microbial profiling using cDNA or even RNA.

## 3 Discussion

With the urgency of accelerating the identification of infectious agents in patients, as well as providing guiding information for treatment, in this study, we presented the conceptual improvement of using RNA as a target molecule for microbial profiling, especially pathogen identification in clinical samples. We argue that active microbes have a higher RNA to DNA ratio than dormant microbes and eukaryotic host cells and that important RNA viral pathogens could also be hidden. With the improvements demonstrated in mtNGS for reducing the overdominance of host genetic material, it can be further improved with the selective removal of host cells using methods such as saponin treatment.^[^ ^27^, ^28^, ^29^ ^]^ In addition, genes with a high number of transcripts, such as 16S and 23S for bacteria and 18S for fungi, which are commonly used for taxonomic analysis, are further elevated in terms of the read ratio in mtNGS and increase the power to detect key microbes. Simultaneously, mtNGS showed a higher efficiency in detecting viruses, in particular RNA viruses that were neglected in previous DNA‐targeted sequencing, which is critical for preparing for the emergence of new infections caused by RNA viruses, including coronaviruses and many others. Finally, the improved profiling of antibiotic resistance genes in mtNGS facilitated both the choice of proper treatment and the monitoring of ARG presence and spread, which is currently an important challenge for global health.

Based on the established improvements of mtNGS, we moved on to utilize the ONT platform to further improve the sequencing speed and read length, resulting in mtTGS. Sequencing of cDNA on ONT platforms (mtTGS) further improved the mapping rate of reads against a multimarker database for microbial identification, a feature that we attributed to the longer read length on ONT platforms. The shorter library preparation time and per‐read sequencing output also results in a greatly reduced total time for clinical sample analysis, which has already been demonstrated for the ONT platform; in the lab, RNA extraction can be performed similarly to DNA extraction. Our approach requires only one extra step of reverse transcription, with the remaining library preparation and sequencing remaining unchanged. In addition, ONT currently enables microbial composition analysis “on‐the‐fly”, and real‐time base calling generates sequences for finished reads continuously, using both ONT‐provided EPI2ME and custom software. We also primarily tested the ability of direct RNA sequencing and targeted sequencing (or rather, targeted rejection), which are currently only possible with ONT. In both applications, we observed a potential for further improving efficiency; however, those methods require further improvement to be effectively utilized in microbial profiling and clinical diagnosis.

The congruence between sequencing results and clinical diagnosis was improved in our study with the adaptation of mtNGS and mtTGS, yet the pitfall remains that culture‐based methods might themselves be prone to bias, and the main pathogen might not be among the top taxa in sequencing results (Tables S6 and S8, Supporting Information). The issue deepens with regard to ARG identification in clinical samples because culture‐based diagnosis and consequent antibiotic sensitivity tests will only focus on the available cultures, while many ARGs are associated with mobile elements such as plasmids and can quickly transfer between different microbes (especially under selective pressure of antibiotic administration). In contrast, sequencing results can identify a wider spectrum of ARGs present in samples, and additional efforts must be carried out to assert the antibiotic resistance profile of specific pathogens. Thus, in the future, our mtNGS and mtTGS should be combined with refinement databases of more specific pathogens revealed in clinics to further remove interference from nonpathogenic commensals, and associating ARGs with pathogens could improve the precision of the treatment schemes. Moreover, sequencing‐based approaches are a promising approach for the diagnosis of infectious disease because a comprehensive spectrum of potential causes—viral, bacterial, fungal, and parasitic—can be identified by a single assay, even when new, emerging pathogens are concerned such as SARS‐CoV‐2.^[^ ^30^, ^31^, ^32^ ^]^ Being relatively cheap and straightforward, mNGS (mainly using Illumina platforms) is the major sequencing‐based pathogen detection, and mtNGS developed in our study (added reverse transcription) only incurs very limited extra cost. In comparison, TGS platforms such as ONT and Pacbio are still in their early developing stage and relatively costly, especially considering the reduced sequencing outcome. Yet, the current stage of TGS mirrors the beginning era of NGS a decade ago, with its potential being explored in many applicational fronts first and the costs driven down gradually. While sequencing‐based approaches should be considered important but not sole methods for pathogen identification in clinics, many other types of diagnostic data such as immune cell and cytokine profiling, medical imaging, *etc*. merit being combined for consideration in treating infections, especially by accumulating large amounts and diverse types of data and applying machine‐learning methods that are increasingly applied in medical science.

In conclusion, we demonstrated the perspectives of RNA‐targeted sequencing, mtNGS and mtTGS, for microbial profiling, particularly in clinical settings, in which they surpass the current DNA‐based approaches (mNGS) in terms of sequencing efficiency. Further improvement using direct RNA sequencing showed preliminary potential, and with foreseeable maturation of the platforms, we expect it to be utilized in a wider range of applications. Developments in reference databases as well as computational methods are also warranted to fully explore the potential in the future RNA‐oriented clinical diagnosis of pathogenic microbes.

## 4 Experimental Section

### Ethics Statement

This study was approved by Ethical approval no S2019‐266‐02 of the Chinese PLA General Hospital, S2020008 of Peking University Third Hospital, 2019‐KY‐081‐01 of Zhujiang Hospital affiliated with Southern Medical University, and only involved the use of residual samples that were sent to these three hospitals. All subjects in our study provided written informed consent, and these samples were anonymized by the removal of any patient identifiable information.

### Sample Selection and Nucleic Acid Extraction

A total of 62 samples (including nine cerebrospinal fluid samples (CSF), 39 bronchoalveolar lavage fluid samples (BALF), and 14 blood samples) were selected for this study, and all of the 62 samples were sequenced by Illumina platform (both mNGS and mtNGS), 43 samples were sequenced by ONT mtTGS, eleven were sequenced by ONT direct RNA sequencing (Figure S1, Supporting Information). Most of the samples were previously determined to be positive (bacteria, fungi, or viruses) using traditional clinical approaches, including culture, targeted nucleic amplification tests (including cases that were diagnosis with Influenza infections first in throat swabs and then confirmed to developed viremia using PCR assays), and serologic assays. Residual samples were stored at −80 °C until the time of sequencing. RNA and DNA were separately extracted using a QIAamp RNA Blood Mini Kit (Qiagen, 52304, Hilden, Germany) and DNeasy PowerSoil Pro Kit (Qiagen, 47016, Hilden, Germany) following the manufacturer's instructions and eluted in RNase‐free water.

### Illumina Library Preparation and Sequencing

A modified protocol for the KAPA Hyper Prep kit (Roche, KK8504, Basel, Switzerland) was used for metagenome library construction. Without rRNA depletion, a KAPA mRNA Library Preparation kit (Roche, KK8441 and KK8544, Basel, Switzerland) was used for metagenome library construction. Sequencing was performed using NovaSeq (PE150).

### ONT Library Preparation and Sequencing

For ONT cDNA sequencing, first‐strand cDNA was synthesized in a 20‐µL reaction mixture with 13 µL of purified RNA from each sample and 100 pmol of primer Rrm (5’‐GACCATCTAGCGACCTCCAC‐NNNNNN‐3’), as previously described.^[^ ^33^, ^34^ ^]^ For double‐strand cDNA synthesis, 100 pmol of primer Rrm and Klenow fragment (3.5 U µL^−1^; Takara, 2140B, Shiga, Japan) were added. Random amplification was conducted with 8 µL of the double‐stranded cDNA template in a final reaction volume of 200 µL, which contained 4 × 10^−6^ m primer Rm (5’‐GCCGGAGCTCTGCAGAATTC‐3’), 90 × 10^−6^ m dNTPs each, 80 × 10^−6^ m Mg^2+^, 10x buffer, and 1 U of KOD‐Plus DNA polymerase (Toyobo, KOD‐201, Osaka, Japan). The amplification product was purified using agarose gel electrophoresis and a QIAquick Gel Extraction Kit (Qiagen, 28604, Hilden, Germany). MinION cDNA library preparation was performed according to the manufacturer's instructions for native barcoding kit (ONT, EXP‐NBD104 and EXP‐NBD114, Oxford, UK) and genomic DNA ligation kit (ONT, SQK‐LSK109, Oxford, UK). When multiplexing, every nine or ten cDNA samples were pooled together. ONT MinKNOW software (v20.06.4) was used to collect raw sequencing data, and Guppy (v4.0.15) was used for real‐time base calling of the raw data. Before ONT direct RNA sequencing, poly(A) tails were added to the purified RNA in a reaction containing 30 µL purified RNA (10–200 ng RNA), 4 µL 10 x *E. coli* Poly(A) Polymerase Reaction Buffer, 4 µL 10 × 10^−3^ m adenosine triphosphate (ATP), 1.5 µL *E. coli* Poly(A) Polymerase (NEW ENGLAND BioLabs, M0276L, MA, USA), and 1 µL RNase Inhibitor (Promega, N2515, WI, USA). The reaction was incubated at 37 °C for 30 min. Then, direct RNA library preparation was performed according to the manufacturer's instructions for direct RNA sequencing (ONT, SQK‐RNA002, Oxford, UK).

### Targeted Nanopore Sequencing with Readfish and UNCALLED

Standard DNA ligation sequencing libraries (ONT, LSK‐109, Oxford, UK) were prepared from cDNA as described above. For Readfish, the host genome reference (Ensembl, GRCh38) index using minimap2 was first constructed.^[^ ^35^ ^]^ Then, the “readfish targets” command was started after the first mux scan during the sequencing by MinKNOW (v3.6.3) and Guppy basecaller server (v3.1.5). The experimental type as host depletion (multi_on = unblock; multi_off = proceed; single_on = unblock; single_off = proceed; no_map = proceed; no_seq = proceed) was also set in the TOML file. Sequencing runs utilized a MinION with four NVIDIA Quadro GV100 GPUs by remote basecall. For UNCALLED, human cDNA and noncoding RNA (Ensembl, GRCh38) were used to build a burrows‐wheeler alignment (BWA) index as a reference to reduce the alignment time. Then, real‐time ReadUntil was started using the command “uncalled real‐time” with the parameter “–deplete” to eject reads mapping to the reference before the sequencing run. The sequencing runs utilized a MinION with a 16‐core central processing unit (CPU) for UNCALLED.

### Data Analysis

Illumina raw reads were processed using Kneaddata (v0.7.4) to trim and filter low‐quality sequences, as well as contaminate human reads with the human genome reference (Hg 38). Profiling the composition of microbial communities was performed using MetaPhlAn2 (v2.7.5) by mapping reads to clade‐specific markers, SILVA‐provided comprehensive, quality checked, and regularly updated datasets of aligned small (16S) and large subunit (23S) ribosomal RNA (rRNA) sequences for bacteria, and 18S rRNA sequences for fungi. Bacterial annotation names were extracted from the bed files, and when the genus name existed, the bacterial name was a combination of the family name and the genus name, separated by an underscore, and if the genus name did not exist, the last annotation classification level was taken. The virus composition was determined by aligning the reads to the reference genome in the National Center for Biotechnology Information (NCBI) virus genome database. For ONT sequencing data analysis, adaptor and barcode sequences of ONT sequencing raw data were trimmed using Porechop (v0.2.3) with default parameters. Trimmed reads were analyzed using minimap2 to identify the 16S, 23S, MetaPhlAn, fungi and virus composition, which aligned reads to the reference genome in the Silva, MetaPhlAn2, and NCBI virus genome databases, respectively. Supplementary and secondary mapping reads from the bam file were removed, and the count matrix of the mapping reads was calculated using the “samtools view” and the “samtools idxstats” parameters, respectively. For ARG detection, the resistance gene identifier is used to predict resistomes from protein or nucleotide data based on homology and single nucleotide polymorphism (SNP) models. The application uses reference data from the CARD, a rigorously curated collection of characterized, peer‐reviewed resistance determinants and associated antibiotics organized by the Antibiotic Resistance Ontology, and AMR gene detection models.

### Statistical Analysis

All statistics were performed in R (v3.6.1). Relative abundance was calculated as the number of reads mapped to pathogens or other microorganisms divided by the overall number of reads for each sample, followed by a log10 transformation. The relevant sample sizes are described in the corresponding figure legends and tables. Comparisons between two groups were performed using the Mann–Whitney–Wilcoxon rank sum test, while comparisons among three or more groups were performed using the Kruskal–Wallis test. The correlation coefficient *r* ^2^ and *p* values between the relative abundance measured by different sequencing platforms were determined by the stat_cor function in the ggpubr package using a linear model. The Spearman correlation coefficient rho between the percentage of SARS‐CoV‐2 mapped reads and the viral load of SARS‐CoV‐2 was determined by the stat_cor function in ggpubr package. Two‐tailed *p*‐values <0.05 were considered significant unless otherwise stated. Venn diagrams were drawn using the VennDiagram package to compare data from different sequencing platforms. Heatmaps was plotted using the ComplexHeatmap package. The R scripts and associated data used in this study, as well as the bacterial annotation bed files, have been uploaded to github (https://github.com/gaimingle10/PathIden).

## Conflict of Interest

The authors declare no conflict of interest.

## Author contributions

N.Z., J.C., J.X., B.L., B.L., and D.C. contributed equally to this work. J.W., H.Z., and L.X. designed the study. J.W., N.Z. and J.C. wrote the paper. B.L., B.L., and D.C. collected the clinical samples. N.Z., W.Z., Y.Z., and X.Z. facilitated multiplatform sequencing. J.C., J.X., B.X., and L.C. analyzed the sequencing data. Z.D., K.W., F.X., K.X., and W.Y. helped the collection of clinical samples.

## Supporting information

Supporting Information

Supplemental Table 1

Supplemental Table 2

Supplemental Table 3

Supplemental Table 4

Supplemental Table 5

Supplemental Table 6

Supplemental Table 7

Supplemental Table 8

Supplemental Table 9

Supplemental Table 10

## Data Availability

The data that support the findings of this study are openly available in GitHub at https://github.com/gaimingle10/PathIden.
