# Supplementary material for: Targeting RNA with Next‐ and Third‐Generation Sequencing Improves Pathogen Identification in Clinical Samples
Source: Adv Sci (Weinh). 2021 Oct 23;8(23):2102593. doi: 10.1002/advs.202102593 (PMC8655164; doi:10.1002/advs.202102593)
Supplement: Supplementary file 1 — Supporting Information [file ADVS-8-2102593-s009.pdf]

Supplementary figures

Targeting RNA with next- and third-generation sequencing improves pathogen identification in clinical samples

Na Zhao[1#], Jiabao Cao[1,2#], Jiayue Xu[1#], Beibei Liu[3#], Bin Liu[4#], Dingqiang Chen[5#], Binbin Xia[1,2], Liang Chen[1], Wenhui Zhang[1,2], Yuqing Zhang[1,2,] Xuan Zhang[1], Zhimei Duan[4], Kaifei Wang[4], Fei Xie[4], Kun Xiao[4], Wei Yan[3], Lixin Xie[4$], Hongwei Zhou[5$], Jun Wang[1,2 $]

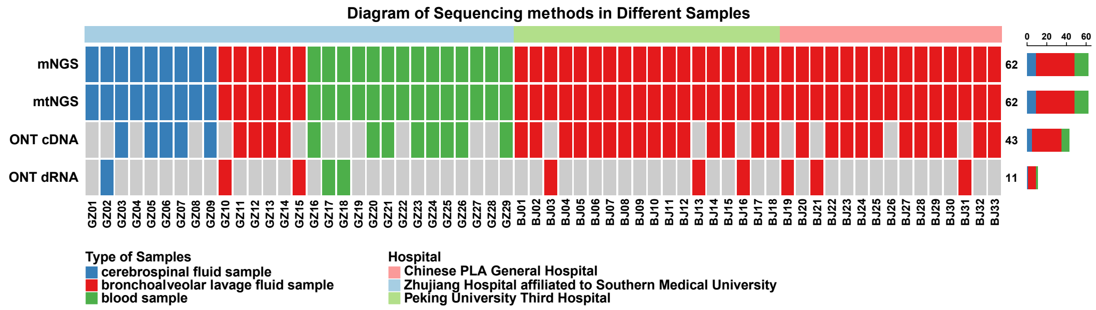

Supplementary figure 1. Diagram of sequencing performed for samples in this study.

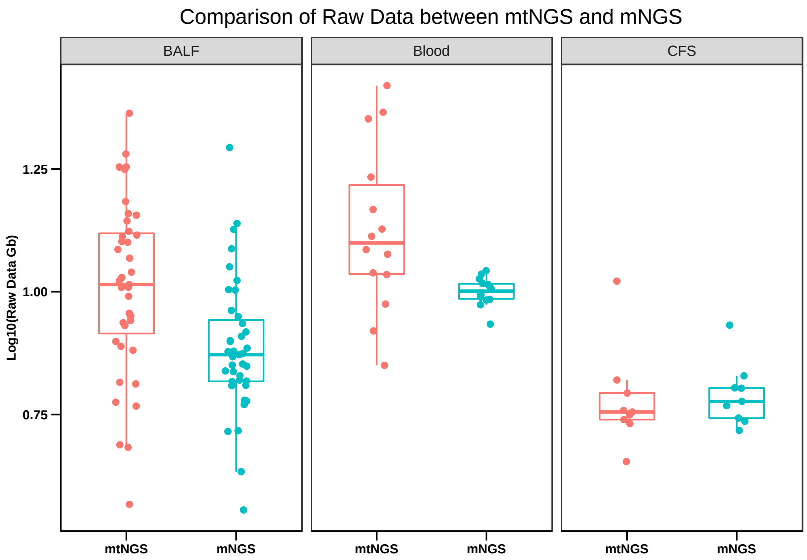

Supplementary figure 2. Comparison of base number in raw data between mtNGS and mNGS.

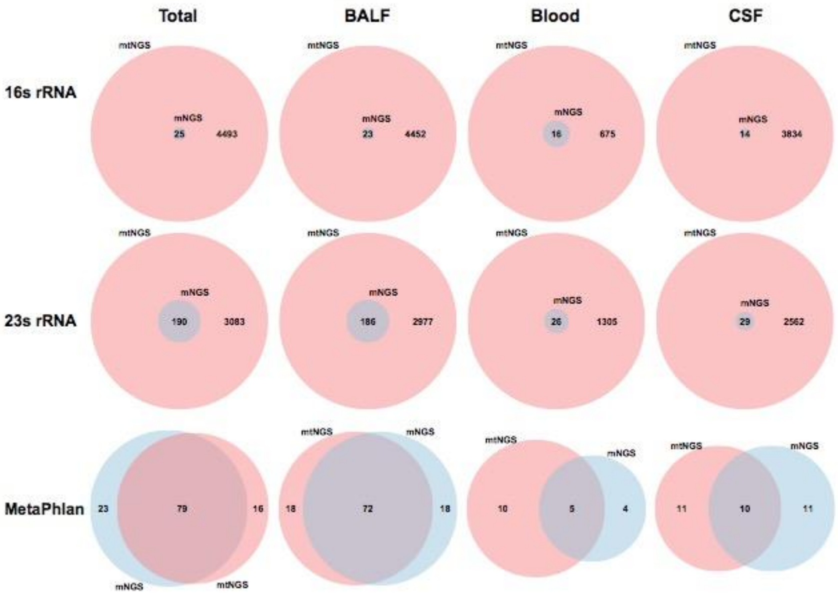

Supplementary figure 3. Venn diagram illustrating the overlap of bacterial composition between mNGS and mtNGS by using 16S genes, 23S genes and MetaPhlAn gene markers as surrogate.

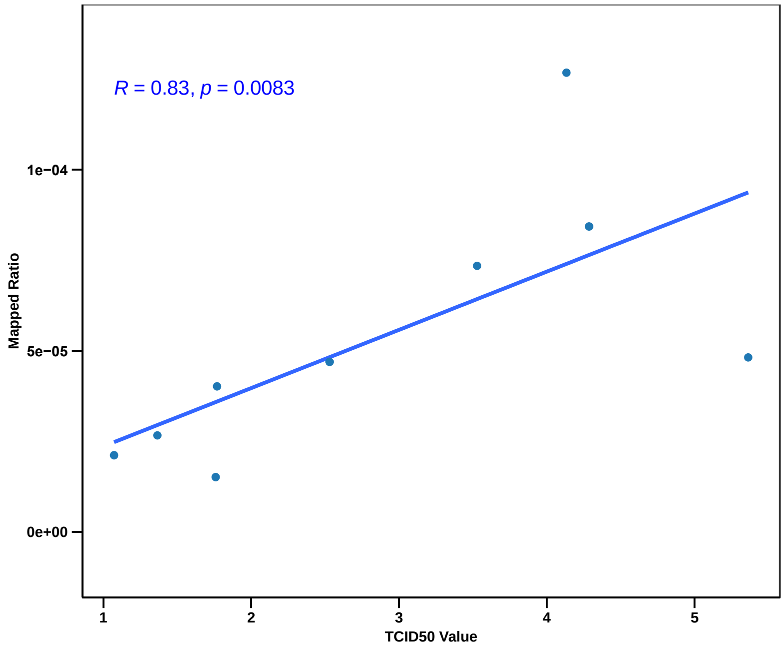

Supplementary figure 4. Correlation of mapped reads ratio of ONT direct RNA sequencing with the viral load of SARS-CoV-2 as determined by RT-PCR in our tested COVID-19 patients. The grey region represents the 95% CIs of linear regression.

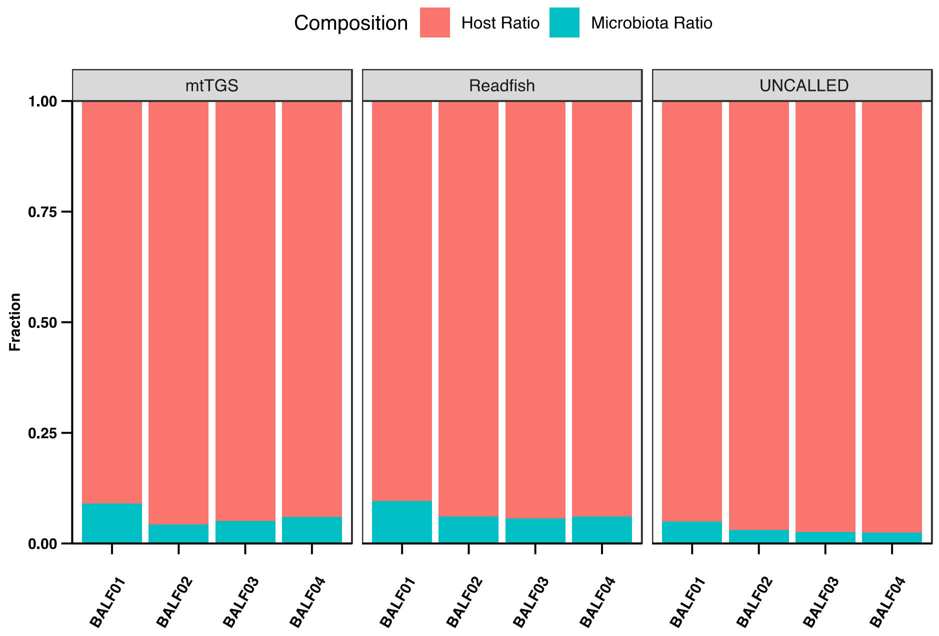

Supplementary figure 5. Composition of microbial and host reads using mtTGS and targeted sequencing using readfish and UNCALLED. Readfish increased slightly the percentage of microbial reads.
